# Early detection of Pre-XDR TB with line probe assay in a high TB burden country

**DOI:** 10.4314/ahs.v21i3.2

**Published:** 2021-09

**Authors:** Laura Madukaji, Isaac Okohu, Saheed Usman, Uche Oyedum, Abdullah Enagi, Abubakar Usman, AS Adedeji, Femi Owolagba, Eke Ofuche, Jay Osi Samuels, Toyin Jolayemi, Prosper Okonkwo

**Affiliations:** 1 APIN Public Health Initiatives Abuja, Nigeria; 2 Bingham University Nigeria; 3 Federal University of Technology Minna, Nigeria

**Keywords:** Pre-XDR TB, line probe assay. in a high TB burden country

## Abstract

**Background:**

Worldwide, tuberculosis (TB) is one of the top 10 causes of death. Drug resistant tuberculosis has lately become a major public health problem that threatens progress made in Tuberculosis (TB) care and control worldwide. The aim of this study was to determine the prevalence of Pre-extensive drug resistant TB among MDR TB in North Central of Nigeria.

**Methods:**

This study was conducted from October, 2018 to August, 2019 with 150 samples. In Nigeria, guidelines for DR-TB as recommended by WHO is followed. All the samples from the patients who gave their consent were transported to a zonal reference TB laboratory (ZRL).

**Results:**

Mean age was 38.6 ± 13.4 years with peak age at 35–44. Out of these 103 samples processed with LPA, 101(98%) were rifampicin resistant and 2 were rifampicin sensitive, 99(96%) were INH resistant and 4 (4%) were INH sensitive, 5(5%) were fluoroquinolone resistant, 98(95%) were fluoroquinolone sensitive, 12 (12%) were Aminoglycoside + Capreomycin resistant, 91(83%) were Aminoglycoside + Capreomycin sensitive.

**Conclusion:**

Multidrug resistant TB and its severe forms (Pre-extensive & extensively drug resistant TB) can be detected early with rapid tool- Line Probe Assay rapid and prevented timely by early initiation on treatment.

## Introduction

Before the discovery of Mycobacterium by Robert Koch in 1882, tuberculosis was seen as disease of the vampires. Tuberculosis (TB) is an infectious disease caused by a bacterium called *Mycobacterium tuberculosis* (MTB). It widely affects the lungs but can also affect other parts of the body. Signs and symptoms of TB may relate to the organ that is affected. However the general signs and symptoms of Pulmonary TB (TB that affects the lungs) and extra pulmonary TB (TB that affects other parts of the body) are fever, chills, night sweats, loss of appetite, weight loss, and fatigue. Because TB is an airborne, its transmission rate is high. However it is preventable, treatable and curable.

World Health Organization (WHO) estimated that one third of the world's population approximately 2 billion people are infected with *Mycobacteria tuberculosis*^1^. Effective treatment of active TB is achieved with combinations of several antibiotics. Drug resistance of tuberculosis (TB) develops when TB survives in the presence of drug therapy^2^.

TB developed resistance to first line therapies that existed since 1950 and thus resulted to the growing emergence of Multidrug-resistant (MDR), Pre-extensive drug-resistant (Pre-XDR) and extensively drug-resistant (XDR) TB. Multidrug-resistant TB (MDR-TB) is TB that does not respond to at least isoniazid and rifampicin, the 2 most powerful anti-TB drugs. Pre-extensive and extensively drug resistant tuberculosis are severe forms of Multidrug resistant tuberculosis. Many factors contribute to anti TB drug resistance including incomplete and inadequate treatment, non-adherence to treatment, person to person transmission, ineffective drug formulation, virulence of the organism, multidrug transporters, host genetic factors, and HIV infection. However in another study, it was revealed that XDR TB resulted majorly by molecular means and the researchers suggested that acquisition of resistance rather than transmission accounts for high prevalence of multidrug resistance.^3^ Mode of drug resistance by the bacterium include production of drug modifying enzymes, barrier mechanisms and drug inactivating enzymes, drug extrusion mechanisms, enhanced efflux and drug Target (gene mutations altering the shape & functions thereby modifing target, over-expression of gene).

It is estimated that 3.9% of all new TB cases and 21% of previously treated TB cases developed MDR-TB in 2015 globally and approximately 250,000 out of the cases (580,000) died from the disease. 4 Nigeria was listed by WHO as one of the countries with a high burden of TB, TB/HIV, MDR TB. With a notification rate of 103.9 per 100,000 population and a with declined mortality rate, TB and MDR TB incidence still remains high. DR TB patients in Nigeria who comprise of new cases (65%) and retreatment cases (88%) were placed on treatment. 5 The prompt detection/management of the patients and other factors resulted to 77% successful treatment rate. The World Health Organization (WHO) in 2008 approved Line Probe Assay (LPA) as a rapid test for the diagnosis of tuberculosis and Rifampicin resistance. In May 2016, WHO issued new recommendations on the use of LPA to detect resistance to second-line anti-TB drugs (SLLPA) to help in placement of patients on shorter MDR TB regimen. This recommendation was after Foundation for Innovative Diagnostics presented a positive outcome of projects carried out in South Africa using Line Probe Assay. In 2018, Nigeria started the second line LPA for eligibility testing for shorter MDR TB regimen.

## Aim & Objectives

The aim of this study was to determine the prevalence of Pre-extensive drug resistant TB among MDR TB in North Central of Nigeria with the objectives to:

Collect samples, process and analyze

Study the susceptibility/resistance pattern of the anti TB drugs

## Research Questions

Will the prevalence of Pre-XDR TB be as high as MDR TB?

What will the pattern of Pre-XDR TB resistance be for different age groups?

## Methodology Study design and study population

This was a prospective study of prevalence of Pre-extensive drug resistant tuberculosis among MDR-TB patients from 5 Drug Resistant TB treatment centres in North central zone of Nigeria using Line Probe Assay (Hain Lifescience, Nehren, Germany). This study was conducted from October, 2018 to August, 2019 with 150 samples. In Nigeria, guidelines for DR-TB as recommended by WHO is followed. All the samples from the patients who gave their consent were transported to a zonal reference TB laboratory (ZRL). The zonal reference laboratory is a recognized for its contribution to national/international TB researches as well as to national TB control and prevention. The National TB Control Program mapped states that feed into this zonal reference laboratory, consequently samples are shipped from the different states to ZRL. It runs microscopy, Phenotypic (Lowenstein-Jensen & liquid culture/DST) and genotypic culture (Genexpert, LPA, fluorotype, Genedrive). All MDR-TB patients both female and male of all age groups that were Rifampicin resistant by Genexpert at the first point of testing were included. Patients who gave consent were included. All LPA results that were invalid and did not correspond with conventional culture were excluded. All contaminated cultures were also excluded. Permission to conduct the study using the treatment centers was granted by the National TB program. Written informed consent was obtained from all patients at the time of enrolment into the study.

Conventional LJ drug susceptibility testing (DST) Conventional 1% proportion phenotypic drug susceptibility testing (DST) on Lowenstein Jensen (LJ) medium was performed. All samples were tested for resistance to the two major first line anti TB drugs -isoniazid (0.2ug/ ml), rifampin (40ug/ml) and second line drugs- kanamycin (20ug/ml), ofloxacin (2ug/ml), amikacin (30ug/ml), capreomycin (40ug/ml). All procedures were carried out according to the National Standard operating procedure (SOP) for LJ Culture/DST

## LPA

LPA is based on PCR amplification of rpo B (RIF), katG and the promoter of inhA (INH), gyrA and gyrB (FLQ), and rrs and the promoter of eis (KAN, AMK, CAP) and subsequent detection of mutations through hybridization to probes immobilized to a nitrocellulose membrane. 6

## DNA extraction for LPA

A chemical DNA extraction method (Genolyse kit from Hain Lifescience, Germany) was used to extract DNA from the concentrate. Manufacturer's instruction was strictly followed to get the DNA extracts. Polymerase chain reaction (PCR) was performed using using 35 µL of primer nucleotide mix, 10 µL of Taq DNA polymerase-PCR buffer mix and 5 µL of supernatant in a final volume of 50 µL. Amplification was done in a thermal cycler. Reverse hybridization was performed using Twincubator. Genotype MTBDRplus kit instruction was strictly followed to find out any deletion in wild-type gene loci and mutation in rpoB (RNA polymerase B subunit), Kat G (catalase peroxidase) and inhA (inoyl coenzyme A reducatse) loci.

## Amplification and hybridization

Amplification was done following preparation of MTBDR plus and MTBDRsl primers and addition of DNA extracts. 10ul of Amplification mix A and 35ul of Amplification mix B (Hains), 5ul of molecular grade water, 5ul of sample (DNA extract). All LPA procedures including amplification and hybridization for direct clinical specimens were performed according to the manufacturer's instructions and National SOP.

## Quality Control

M. tuberculosis H37Rv was used as quality control (QC) for both LJ Culture/DST and LPA methods. The QC strain was a known pan susceptible strain and replicated in the results of the methods used in this study.

## Data analysis

Data were analysed with Graph pad prism (version 3) & Statistical Package for Social Sciences (SPSS) versions 24.0. The proportion of Pre-XDR TB among the study population was calculated. All significant variables were analysed with results expressed as odds ratios (OR) with 95% confidence Interval (CI). For all statistical analysis, a p value of < 0.05 was considered significant.

## Results

A total of 150 patients that met the inclusion criteria for selection of study population were enrolled. We excluded 6 contaminated conventional LJ cultures and 12 invalid LPA and analyzed 103 results. One hundred and three LPA results that corresponded with conventional LJ culture/DST were analyzed. Table 1 represents the characteristics of the patients. Out of 103 patients, the frequency (%): sex-female was 53 (51.5) and frequency (%) sex: male was 50 (48.5). The mean age was 38.6 ± 13.4 years with peak age at 35–44.

**Table 1:** Characteristics of study population

Gender	Frequency (%)
Male	50
Female	53
**Age**	
15–24	13
25–34	26
35–44	41
45–54	7
≥55	16
**HIV status**	
Positive	39
Negative	71
**Location**	
FCT	10.7
Benue	39.8
Kogi	21.4
Nasarawa	19.4
Niger	8.7

Out of these 103 samples processed with LPA, 101(98%) were rifampicin resistant and 2 were rifampicin sensitive, 99(96%) were INH resistant and 4 (4%) were INH sensitive, 5(5%) were fluoroquinolone resistant, 98(95%) were fluoroquinolone sensitive, 12 (12%) were Aminoglycoside + Capreomycin resistant, 91(83%) were Aminoglycoside + Capreomycin sensitive. With LJ proportional DST, all the 103(100%) were rifampicin resistant and INH resistant, 7 (7%) were fluoroquinolone resistant, 96(93%) were fluoroquinolone sensitive, 9(9%) were Aminoglycoside + Capreomycin resistant, 94(91%) were Aminoglycoside + Capreomycin sensitive (Table 2).

**Table 2:** Concordance between LPA and culture

LPA	N (%)	LJ proportion DST	N (%)
RIF resistant	101 (98)	RIF resistant	103 (100)
RIF sensitive	2 (2)	RIF sensitive	0 (0)
INH resistant	99 (96)	INH resistant	103 (100)
INH sensitive	4 (4)	INH sensitive	0 (0)
FLQ resistant	5 (5)	FLQ resistant	7 (7)
FLQ sensitive	98 (95)	FLQ sensitive	96 (93)
AG + Cap resistant	12 (12)	AG + Cap resistant	9 (9)
AG+ Cap sensitive	91(83)	AG + Cap sensitive	94 (91)

RIF-Rifampicin, INH-Isoniazid, FLQ-Fluoroquinolone, AG+Cap-Aminoglycoside + Capreomycin

Five (5%) of the 103 patients were resistant to fluoroquinolone, 4% were resistant to KAN/AMK/CAP, 11% were resistant to KAN/CAP/VIO, 1% showed resistance to KAN/AMK/CAP/VIO, 78% were pan susceptible to both fluoroquinolone and injectables and 100% were resistant to both Isoniazid and rifampicin. Twenty-one cases of Pre-XDR TB were detected, resulting to prevalence of 20.5% (Table 3). Table 4 showed Pre-XDR TB detected among the age groups as follows: 15–24 years (3%), 25–34 years (6%), 35–44 years (8%), 45–54 years (1%) and ≥55 (3%). The frequency of mutation for rpoβ was WT 8, MUT2B (4), MUT 2B(4), WT 7 (5), WT 7, MUT3(4), WT 8, MUT3 (20), WT8 (7), WT 3, WT4 (5), MUT 2B, MUT3 (4), WT (4). inhA was WT (10), Kat G was WT (12) and WT MUT 1(15). Frequency of mutation for second line anti TB drugs were WT1, WT2 (9) and WT1 (4) for rrs; WT 1, WT2 (2), WT1 (1) for eis; WT 1, WT2, WT3 (3), WT3 (2) for gyrA (Table 5).

**Table 3:** Susceptibility Testing Pattern of anti TB drug using LPA

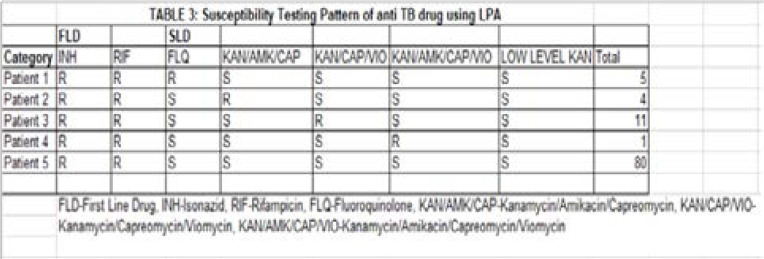

**Table 4:** Age distribution of Pre-XDR TB

	Pattern of resistance (RIF, INH & SLD)				
Age	R,R, R,S,S, S	R,R, S,R,S,S,S	R,R,S,S,R,S, S	R,R, S,S,S,R,S	R,R, S,S,S,S,S
15–24	1	2	0	0	10
25–34	0	2	4	0	20
35–44	3	0	5	0	34
45–54	0	0	1	0	5
≥55	1	0	1	1	11

RIF-Rifampicin, INH-Isoniazid, SLD-Second Line Drug (Fluoroquinolone, Kanamycin/Amikacin/Capreomycin, Kanamycin/Capreomycin/Viomycin, Kanamycin/Amikacin/Capreomycin/Viomycin)

**Table 5:** Regions of mutation observed with LPA

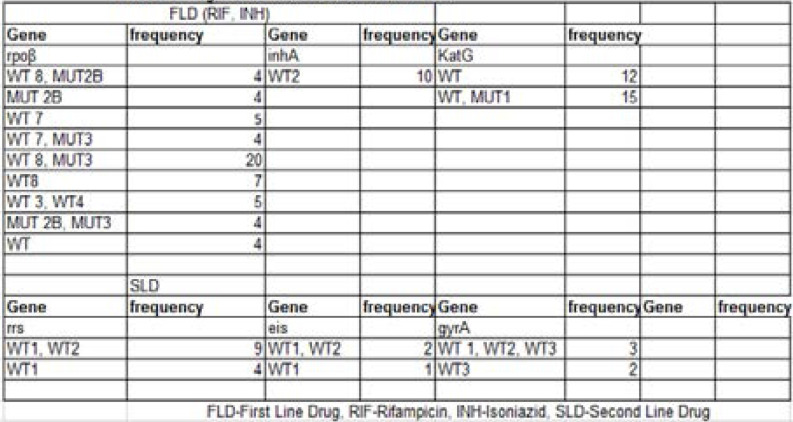

## Discussion

This study was conducted to determine the prevalence of pre-XDR-TB cases among Drug Resistant TB suspects in North Central zone of Nigeria. To confirm the performance of LPA, we assessed its concordance with LJ proportional Drug Susceptibility Testing which is the gold standard. The LPA showed 98% concordance with LJ proportional DST and this agrees with 96% concordance report 14. The DST pattern showed that majority of the pre-XDR TB patients were resistant to the fluoroquinolone and one of the high level injectables (KAN/CAP/VIO) which is similar to a study done by Elisa et al., (2015). The resistance observed with the drugs could be attributed to the direct alteration of rrs, the shared target via the A1401G mutation is known to confer unequivocal cross-resistance to all three drugs 7. The prevalence of 20.5% as revealed in this study is similar to studies done in Nigeria, Bangladesh, South Africa and China where the detection of pre-XDR-TB among MDR-TB patients was 21%, 16.2%, 14.0% and 34% respectively 8–11. It is also consistent with prevalence of 16.7% reported among MDR TB patients in South Western zone of Nigeria 12. Reason for this the high prevalence is presumed to be related to high burden of MDR TB in these countries.

This study reported high rate of prevalence of Pre-XDR TB with age groups 15–44 years which is consistent with other studies done in India, Nigeria and Ethiopia that reported higher rate of pre-XDR-TB cases among age groups 18–25 years, 15–29 years, 15–34 years respectively 12–14. The high rate of this severe form of DR TB in the active age groups might be due to frequent movement, increased outdoor contact and higher case notification due to greater health awareness among this group of adult. In this study, peak age group was found to be 35–44 years which is different from the reports of some studies with a peak age group of 10–25 years and 25–44 years. 15,16 The reason for the difference could be due to the different age intervals chosen in the studies (wide age ranges chosen in the Malaysian and Pakistan studies). However the report of this study is consistent with WHO report of higher frequencies among ages of 25–34 and 35–44 years.^17^

The frequency of mutation for rifampicin conferred by rpoβ was WT 8, MUT2B (4), MUT 2B (4), WT 7 (5), WT 7, MUT3 (4), WT 8, MUT3 (20), WT8 (7), WT 3, WT4 (5), MUT 2B, MUT3 (4), WT (4). Multiple mutations occurred more than single mutations and also more at WT 8, MUT 3. Among various mutations observed using LPA, majority of the drug resistant isolates had multiple mutations for Rifampicin resistance. These multiple mutations are believed to be more probable in high TB incidence places. The frequency of mutation for rifampicin conferred by Kat G and inhA occurred more at WT (12); WT MUT 1(15) and WT (10) respectively. Mutation was seen more with Kat G which is consistent with KatG was WT (12) and WT MUT 1(5). This is similar to the result of studies where most of the isoniazid resistance isolates had mutation in KatG than the in inhA gene. 18–21 The isoniazid resistance commonly associated with KatG has been found to be the case in many high TB burden countries due to ongoing transmission of the strain.

## Conclusion

Nigeria is highly burdened with TB, MDR TB and TBHIV with a high prevalence of Pre-extensive drug resistant TB among the MDR TB group. Multidrug resistant TB and its severe forms (Pre-extensive & extensively drug resistant TB) can be detected early with rapid tool- Line Probe Assay rapid and prevented timely by early initiation on treatment. The gold standard from the range of moderate to high is thus a good screening tool for the rapid detection of resistance to second-line anti-TB drugs in countries with high burden of MDR TB.
